# Obstructed labor and its association with adverse feto-maternal outcome in Ethiopia: a protocol for a systematic review and meta-analysis

**DOI:** 10.1186/s13643-021-01611-x

**Published:** 2021-02-16

**Authors:** Yordanos Gizachew Yeshitila, Melaku Desta, Abraham Kebede

**Affiliations:** 1 School of Nursing, College of Medicine and Health science, Arbaminch University, Arbaminch, Ethiopia; 2 Department of Midwifery, College of Health Science, Debre Markos University, Debre Markos, Ethiopia; 3 University of Brighton, Brighton, England

**Keywords:** Prevalence, Obstructed labor, Adverse outcome, Maternal, Fetal, Ethiopia

## Abstract

**Background:**

Obstructed labor accounted for 22% of obstetrical complications and 9% of all maternal deaths in low- and middle-income countries. Even though there are separate studies regarding obstructed labor and its complication in Ethiopia, their results are inconsistent. The objectives of this review will be to estimate the pooled the prevalence of obstructed labor and to identify adverse feto-maternal outcomes associated with obstructed labor in Ethiopia.

**Methods:**

Preferred Reporting Items for Systematic Reviews and Meta-Analyses guideline will be followed to conduct this systematic review and meta-analysis. The databases we will search will be PubMed, Cochrane Library, Google Scholar, CINAHL, African Journals Online, Dimensions, and Summon per country online databases. To search the relevant literature, we will use the following key search terms: “prevalence,” “adverse outcome,” “obstructed labour,” “maternal near miss,” “neonatal near miss,” “perinatal outcome,” “cesarean section,” “obstetric fistula,” “uterine rupture,” and “Ethiopia.” Joanna Briggs Institute Meta-Analysis of Statistics Assessment and Review Instrument will be used for evaluating the quality of the studies. Appropriate statistical tests will be conducted to quantify the between studies heterogeneity and for the assessment of publication bias. We will check individual study influence analysis and also do subgroup analysis. The STATA version 15 will be used for statistical analysis.

**Discussion:**

Our systematic review and meta-analysis will provide the pooled prevalence of obstructed labor and its association with adverse feto-maternal outcomes in Ethiopia. The finding of this study will be helpful to design appropriate preventive and promotive strategies for reducing of obstructed labor-related maternal mortality.

**Systematic review registration:**

PROSPERO CRD42020196153.

**Supplementary Information:**

The online version contains supplementary material available at 10.1186/s13643-021-01611-x.

## Background

World Health Organization (WHO) defines obstructed labor as strong uterine contractions without descending of the fetus through the pelvis due to obstruction that usually occurs at the pelvic brim, in the cavity, or at the outlet of the pelvis [1].

In 2017, WHO estimated about 295,000 women died during and after pregnancy and childbirth. Of which, 94% occurred in low-resource settings, and most could have been prevented. Sub-Saharan Africa accounted for two thirds of the total global maternal deaths (196,000) [2]. Direct obstetric causes accounted for about 86% of all maternal deaths globally in 2015 [3]. Obstructed labor (OL) is a major public health challenge which remains a significant cause of maternal and perinatal mortality and morbidity, both short and long term. In addition, OL is known to cause a huge economic burden for developing countries [3–5].

Unlike high-income countries where OL accounts for a negligible component of maternal mortality, in low-income countries, it remains a major cause of maternal death [6]. In data from review of 40 low- and middle-income countries in 2017, OL accounted for 22% of obstetrical complications and 9% of all maternal deaths in low- and middle-income countries (LMIC) [7]. In sub-Saharan Africa (SSA), 9% of the total maternal death is attributed to OL [7]. Ethiopia is among the 15 countries considered to be at a “very high alert” or “high alert”, according to the Fragile States Index with a high maternal mortality rate [2]. Furthermore, an estimated 11 to 22% of maternal deaths in Ethiopia are attributed to OL [8, 9].

OL causes significant short- and long-term maternal morbidities which include but are not limited to urological, gynecological, gastrointestinal, musculoskeletal, neurological, or dermatological injury; social isolation; divorce; poverty; malnutrition; depression; and premature mortality [10–13]. The most severe complication of OL is obstetric fistula. About 80–90% of obstetric fistula is caused by OL [7]. In the newborn, OL may cause asphyxia leading to stillbirth (29 to 44%), brain damage, or neonatal death. The fetal case fatality rate is also around 95% [12].

One of the health sector Transformation Plan (HSTP) initiatives of Ethiopia was to reduce MMR to 177 death per 100,000 live birth (LB) in 2020 [14]. Ethiopia is also expected to be in line with the Sustainable Development Goal (SDG) which targeted countries achieving an MMR of 70 per 100,000 live births (LB) by 2030 with no country having an MMR of more than twice the global average [15]. Attaining this goal seems to be ambitious for developing countries including Ethiopia [2]. Studies conducted so far show that the burden of OL and its adverse feto-maternal outcomes are high and a common challenge in Ethiopia [16–23], with the leading cause of maternal mortality associated with OL being uterine rupture [24]. The prevalence of OL in Ethiopia is estimated to be 20% [25]. However, the prevalence varies across different regions, 17.5% in Tigray region [26] and 9.6% in Oromia region [23]. In studies conducted so far, referral time, travel distance, residence, age, antenatal care (ANC) follow-up, gravida, parity, access to transport, height, malnutrition at young age, and age at pregnancy were significantly associated with OL [17, 19, 27–29].

Determining the prevalence of OL, identifying its predictors and investigating the adverse outcomes of OL are very essential for the reduction of OL-related death and morbidities by ascertaining modifiable factors and to inform evidence-based medical and public health interventions. Even though there are some studies, the prevalence of OL and its impact on feto-maternal outcomes were inconsistent across Ethiopia. Additionally, there is limited data regarding OL and its associated adverse feto-maternal outcome at the national level. Therefore, this systematic review and meta-analysis aims to estimate to pooled national prevalence of OL, its predictors, and risk of adverse maternal and perinatal outcomes of OL.

## Methods

### Protocol and registration

This systematic review and meta-analysis will be conducted following the recommendation of the Preferred Reporting Items for Systematic Reviews and Meta-Analysis (PRISMA-P) guidelines. This review protocol is registered in the International Prospective Register of Systematic Reviews (PROSPERO) database and can be accessed at https://www.crd.york.ac.uk/PROSPERO/display_record.php?RecordID=CRD42020196153.

The systematic review protocol is written in accordance with the PRISMA-P guidelines. See Additional file 1 for the completed PRISMA-P checklist.

### Eligibility criteria

#### Study design/characteristics

Observational studies (cross-sectional, case controls, and cohort) that report the prevalence of OL and the association between obstructed and adverse feto-maternal outcomes among mothers or women who have recently given birth will be considered for inclusion. In this review, we will consider studies conducted in Ethiopia and published in English up to August 2020.

### Population

We will include studies on mothers or women who have recently given birth or who have been diagnosed with OL, studies reporting OL as a cause for adverse perinatal outcome/birth outcome, and studies reporting OL and associated feto-maternal outcomes.

### Settings

Studies conducted in Ethiopia will be considered. As of 2020, there are 10 regions and 2 chartered cities in Ethiopia.

### Outcomes of interest

The primary outcome will be the prevalence of OL. We will use definitions according to accepted standardized definition, the term “obstructed labour” indicates a failure to progress due to mechanical problems despite strong uterine contractions—a mismatch between fetal size, or more accurately, the size of the presenting part of the fetus, and the mother’s pelvis, although some malpresentations, notably a brow presentation or a shoulder presentation (the latter in association with a transverse lie) will also cause obstruction [12, 30].

### The secondary outcomes will be

*Maternal adverse outcome*: uterine rupture, sepsis, PPH, vesicovaginal fistula (VVF), bladder rupture, wound dehiscence, anemia, perineal tear, cervical tear, hysterectomy, and maternal death.

*Neonatal adverse outcome*: asphyxia, sepsis, still birth, neonatal jaundice, birth injury (cranial injury), and fetal/neonatal death.

### Information sources and search strategy

A systematic search of PubMed/MEDLINE, Embase, CINAHL, and Cochrane Library will be conducted from inception onwards. The search strategy will be developed in PubMed/MEDLINE (see Additional file 2) and adapted to the other bibliographic databases. Search terms will include subject headings (e.g., MeSH in PubMed/MEDLINE) for each database and free-text words for the key concepts of “prevalence,” “epidemiology,” “incidence,” “adverse outcome,” “obstructed labour,” “labour complication,” “maternal near miss,” neonatal near miss,” “cesarean section,” “obstetric fistula,” “birth asphyxia,” “still birth,” “meconium stained,” and “Ethiopia.” Grey or difficult to locate literature will be searched, including Google Scholar, Open Grey, and the World Health Organization (WHO) websites. We will also search reference lists of included studies and related reviews.

### Study selection and data extraction

The citations will be downloaded into the Endnote software and will exclude duplicate articles. Two review team members (YGY, MD) will independently screen all studies identified from the literature search in two stages. In the first stage, the two reviewers (YGY, MD) will independently screen titles and abstracts based on the eligibility criteria outlined above. They will document, with reasons, the studies excluded from the review. In the second stage, full-text versions of selected articles will be downloaded/retrieved and examined in detail by the two reviewers (YGY, MD) for eligibility. They will extract data from eligible papers identified during the full text screening step. In the event of disagreement, the two authors will confer and discuss with each other and, if necessary, a third review author (AH) to reach consensus. References of all considered articles will be hand searched to identify any relevant report missed in the search strategy. Using the format of the validated standard data extraction form, we will extract the following information: first author, region in which the study was conducted, year of publication, study period, study design, sample size, aim of the study, definition provided by the authors, and study population. Data will be extracted independently by two authors (YGY, MD). In case of missing data, an attempt will be made to contact the corresponding and last authors of studies by email and telephone. If the author fails to provide additional information, a decision will be made as to whether to include the study in the final review. A flow chart showing the studies included and excluded at each stage of the study selection process will be provided.

### Assessment of methodological quality

Domains of the quality of studies retained for full-text review will be checked by authors [YGY and MD] independently. The Joanna Briggs Institute Meta-Analysis of Statistics Assessment and Review Instrument will be used to appraise the quality of included studies. This instrument provides different tools for assessing quality according to study design. For example, the JBI critical appraisal tool has eleven items to assess cohort studies, ten items to assess case-control studies, and eight items to assess analytic cross-sectional studies [31]. See Additional file 3 for details on the Joanna Briggs Institute’s critical appraisal checklists for observational studies. Results of study quality assessment and data extracted will be exported in a preferred format for data synthesis and statistical analysis.

### Data synthesis and statistical analysis

#### Narrative synthesis

We will synthetize primary studies to explore heterogeneity descriptively such as structured narratives or summary tables, measures of prevalence of OL, and its associated feto-maternal outcome. We would expect that differences in studies are unavoidably heterogeneous. If extracted data are insufficient for quantitative synthesis, that is if we find a very high degree of clinical and methodological heterogeneity, we will not pool the results but will instead summarize the results narratively by using tables and figures.

#### Quantitative synthesis

If data are appropriate for quantitative synthesis of primary and secondary outcomes, we will conduct random-effects meta-analysis. STATA version 16 will be used to pool estimates of the prevalence of OL and effect sizes on the effect of OL on feto-maternal outcome across study designs. We will implement the random effects model using the method of DerSimonian and Laird, with the estimate of heterogeneity being taken from the inverse-variance fixed-effect model [32, 33]. For the meta-analysis of prevalence, a procedure in STATA, called metaprop, that performs the Freeman-Tukey double arcsine transformation, will be used to compute the weighted pooled estimate and perform back-transformation on the pooled estimate [34]. The random-effects model will also be used to combine the prevalence estimates. The estimated pooled effects will be reported as proportions for OL with 95% CI and odds ratios with 95% confidence intervals (CIs) for secondary outcomes. The model selection, however, will further be informed by the heterogeneity assessment.

The Higgins I2 statistic will be used to describe the percentage of the total variability in study estimates that is due to heterogeneity. The I2 statistic values of 25%, 50%, and 75% would mean low, medium, and high heterogeneity, respectively [35]. Sources of the between studies heterogeneity will be assessed by a subgroup analysis. The potential variables we will consider for a subgroup analysis include the regions of the study, age of the mother, parity, and study design. To further assess whether these factors explain any observed heterogeneity of effect size estimates, we will conduct a meta-regression. We will also conduct a single study influence analysis to observe the effect of omitting a single study on the overall pooled effect estimate [36].

The publication bias, which represents the tendency to report positive findings [37], will be visually checked by inspecting the funnel plot and also objectively by using the Harbord’s regression test to statistically assess the asymmetry of the funnel plot [38].

## Discussion

The protocol describes a planned systematic review and meta-analysis to determine the prevalence of OL and adverse feto-maternal outcome associated with OL in Ethiopia. Complications secondary to OL can be avoided if a woman in OL is identified early and appropriate action is taken [1]. In low- and middle-income countries (LMIC), provision of rapid access to comprehensive obstetrical emergency care is consequently crucial to mitigate these burdens of morbidity and mortality due to OL [11].

We believe findings from this systematic review and meta-analysis could provide important evidence for health policy formulation and preventive programs, which could help in reduction of OL-related death and morbidities by ascertaining modifiable factors, and subsequently, attaining Sustainable Development Goals (SDGs) (2, 3, and 4) [39].

The proposed systematic review and meta-analysis will be reported in accordance with the reporting guidance provided in the Preferred Reporting Items for Systematic Reviews and Meta analyses (PRISMA) statement [40] and the Meta-analysis Of Observational Studies in Epidemiology (MOOSE) reporting guideline [41]. Any changes made to this protocol when conducting the study will be outlined in PROSPERO and reported in the final manuscript. Results will be disseminated through conference presentations and publication in a peer-reviewed journal.

### Strength and limitation of this study

- Available studies will be carefully assessed for quality using JBI Critical Appraisal Checklist.
- This study will systematically provide evidence on effect of OL on feto-maternal outcome.
- Possible limitations of this study may be related with our anticipation of identifying observational studies conducted with different study designs, populations, and contexts and with a variable quality of reporting of methods and results.
- In addition, publication and reporting biases can misrepresent the planned review and meta-analysis.

## Supplementary Information

**Additional file 1.** PRISMA-P checklist.

**Additional file 2.** Search strategy.

**Additional file 3.** JBI Critical Appraisal Checklist.

## Data Availability

Not applicable.

## Abbreviations

CI: Confidence interval
JBI: Joanna Briggs Institute
LB: Low birth
LMIC: Low- and middle-income countries
MOOSE: Meta-analysis Of Observational Studies in Epidemiology
MMR: Maternal mortality rate
OL: Obstructed labor
PPH: Post-partum hemorrhage
PRISMA-P: Preferred Reporting Items for Systematic Reviews and Meta-analysis
SDGs: Sustainable Development Goals
WHO: World Health Organization
