# Supplementary material for: Obstructed labor and its association with adverse feto-maternal outcome in Ethiopia: a protocol for a systematic review and meta-analysis
Source: Syst Rev. 2021 Feb 16;10:57. doi: 10.1186/s13643-021-01611-x (PMC7887789; doi:10.1186/s13643-021-01611-x)
Supplement: Supplementary file 2 — Additional file 2. Search strategy. [file 13643_2021_1611_MOESM2_ESM.docx]

Search strings built for literature search in the respective databases for a systematic review and meta-analysis on the obstructed labor and its association with adverse feto-maternal outcome in Ethiopia: A protocol for systematic review and meta-analysis.”

Key concepts: obstructed labour, adverse outcome, maternal, fetal, Ethiopia

(((((((((((((((("Obstructed labour"[All Fields] OR ("dystocia"[MeSH Terms] OR "dystocia"[All Fields] OR ("abnormal"[All Fields] AND "labour"[All Fields]) OR "abnormal labour"[All Fields])) OR ("dystocia"[MeSH Terms] OR "dystocia"[All Fields] OR ("labour"[All Fields] AND "dystocia"[All Fields]) OR "labour dystocia"[All Fields])) OR "Labour abnormality"[All Fields]) OR ("maternal mortality"[MeSH Terms] OR ("maternal"[All Fields] AND "mortality"[All Fields]) OR "maternal mortality"[All Fields])) OR ("labour complication"[All Fields] OR "obstetric labor complications"[MeSH Terms] OR ("obstetric"[All Fields] AND "labor"[All Fields] AND "complications"[All Fields]) OR "obstetric labor complications"[All Fields] OR ("labor"[All Fields] AND "complication"[All Fields]) OR "labor complication"[All Fields])) OR "Maternal near-miss"[All Fields]) OR "Neonatal near miss"[All Fields]) OR ("perinatal mortality"[MeSH Terms] OR ("perinatal"[All Fields] AND "mortality"[All Fields]) OR "perinatal mortality"[All Fields] OR "perinatal death"[MeSH Terms] OR ("perinatal"[All Fields] AND "death"[All Fields]) OR "perinatal death"[All Fields] OR ("perinatal"[All Fields] AND "mortality"[All Fields]))) OR ("fistula"[MeSH Terms] OR "fistula"[All Fields])) OR ("caesarean section"[All Fields] OR "cesarean section"[MeSH Terms] OR ("cesarean"[All Fields] AND "section"[All Fields]) OR "cesarean section"[All Fields])) OR ("uterine rupture"[MeSH Terms] OR ("uterine"[All Fields] AND "rupture"[All Fields]) OR "uterine rupture"[All Fields])) OR "Instrumental delivery"[All Fields]) OR "Operative delivery"[All Fields]) OR "Prolonged labor"[All Fields]) OR "Adverse birth outcome"[All Fields]) AND ((((("maternal death"[MeSH Terms] OR ("maternal"[All Fields] AND "death"[All Fields]) OR "maternal death"[All Fields]) OR "Adverse maternal outcome"[All Fields]) OR ("perinatal death"[MeSH Terms] OR ("perinatal"[All Fields] AND "death"[All Fields]) OR "perinatal death"[All Fields])) OR "Adverse perinatal outcome"[All Fields]) OR "Feto-maternal outcomes"[All Fields])) AND ("ethiopia"[MeSH Terms] OR "ethiopia"[All Fields])
